# Overexpression of *Medicago SVP* genes causes floral defects and delayed flowering in *Arabidopsis* but only affects floral development in *Medicago*

**DOI:** 10.1093/jxb/ert384

**Published:** 2013-11-18

**Authors:** Mauren Jaudal, Jacob Monash, Lulu Zhang, Jiangqi Wen, Kirankumar S. Mysore, Richard Macknight, Joanna Putterill

**Affiliations:** ^1^Flowering Laboratory, School of Biological Sciences, University of Auckland, Private Bag, Auckland 92019, New Zealand; ^2^Department of Biochemistry, University of Otago, PO Box 56, Dunedin 9054, New Zealand; ^3^Plant Biology, Samuel Roberts Noble Foundation, Ardmore, OK 73401, USA

**Keywords:** *Arabidopsis*, flowering, legume, MADS, *Medicago*, *SVP*.

## Abstract

The MADS-domain transcription factor SHORT VEGETATIVE PHASE plays a key role as a repressor of the transition to flowering and as a regulator of early floral development in *Arabidopsis thaliana* (*Arabidopsis*). However, no flowering-time repressors have been functionally identified in the model legume *Medicago truncatula* (*Medicago*). In this study, phylogenetic analysis of two closely-related *MtSVP-like* sequences, *MtSVP1* and *MtSVP2*, showed that their predicted proteins clustered together within the eudicot SVP clade. To determine if the *MtSVP-like* genes have a role in flowering, they were functionally characterized in *Medicago* and *Arabidopsis*. Transcripts of both *MtSVP* genes were abundant and broadly expressed in vegetative tissues but were detected at much lower levels in flowers in *Medicago*. Over-expression of the *MtSVP* genes in *Arabidopsis* resulted in delayed flowering and flowers with many abnormal phenotypes such as leafy sepals, changes to floral organ number and longer pedicels than the wild type. By contrast, in transgenic *Medicago*, over-expression of *MtSVP1* resulted in alterations to flower development, but did not alter flowering time, suggesting that *MtSVP1* may not function to repress the transition to flowering in *Medicago.*

## Introduction

Plants precisely regulate the timing of the transition from vegetative to reproductive growth to optimize sexual reproduction and productivity (Putterill *et al.*, 2004). The genetic pathways that regulate flowering time have been investigated in many plants, but are best understood in the monocot cereals such as rice, wheat, and barley and in the eudicot model plant, *Arabidopsis thaliana* (*Arabidopsis*) (Trevaskis *et al.*, 2006; Kim *et al.*, 2009; Srikanth and Schmid, 2011; Andres and Coupland, 2012). In *Arabidopsis*, six major pathways influence flowering time in response to external (photoperiod, vernalization, and thermosensory pathways) and endogenous signals (autonomous, age, and gibberellin) with carbohydrate status also playing a critical role in controlling the transition to flowering (Wahl *et al.*, 2013). Flowering signals from these multiple pathways are integrated by floral integrator genes including *FLOWERING LOCUS T (FT), TWIN SISTER OF FT (TSF)*, and *SUPPRESSOR OF OVER-EXPRESSION OF CONSTANS1* (*SOC1*) that go on to promote the transition to flowering.

In *Arabidopsis*, the gene encoding the MADS-domain transcription factor SHORT VEGETATIVE PHASE (AtSVP) is expressed broadly during vegetative development including in leaves and shoot apices and functions as a flowering time repressor (Hartmann *et al.*, 2000; Lee *et al.*, 2007*b*). Consistent with a role in flowering time regulation, *Arabidopsis svp* mutants are early flowering (Hartmann *et al.*, 2000) while overexpression of *SVP* (*35S:AtSVP*) leads to a late-flowering phenotype (Masiero *et al.*, 2004; Li *et al.*, 2008). AtSVP functions, in part, by complexing with a second important flowering time repressor, the MADS-domain protein FLOWERING LOCUS C (FLC) and delays flowering by repressing *FT, TSF*, and *SOC1* in the leaves and *SOC1* at the apical meristem (Searle *et al.*, 2006; Lee *et al.*, 2007*a*; Fujiwara *et al.*, 2008; Li *et al.*, 2008; Jang *et al.*, 2009). The expression of *AtSVP* is down-regulated by the thermosensory, autonomous, and gibberellin flowering-time pathways and after the transition to flowering there is loss of *AtSVP* expression in the inflorescence meristem (Hartmann *et al.*, 2000; Lee *et al.*, 2007*b*; Liu *et al.*, 2007; Li *et al.*, 2008).

However, *AtSVP* expression is detectable in floral primordia at the very earliest stages of their development (Hartmann *et al.*, 2000; Liu *et al.*, 2007). AtSVP together with the closely-related MADS-domain protein AGAMOUS-LIKE 24 (AGL24) and a third MADS factor SOC1 play redundant roles in promoting floral primordium proliferation during stage 1 and stage 2 of flower development by repressing the class E MADS domain floral homeotic gene, *SEPALLATA 3* (*SEP3*) (Gregis *et al.*, 2009; Liu *et al.*, 2009). AtSVP and AGL24 also form heterodimers with the MADS domain protein APETALA1 (AP1) to repress class B and C floral organ identity genes via recruitment of the SEUSS-LEUNIG co-repressor complex (Gregis *et al.*, 2009; Posé *et al.*, 2012). After stage 2, *AtSVP* expression is repressed by SEP3, which then functions with LFY to activate the class B and class C genes (Kaufmann *et al.*, 2009; Liu *et al.*, 2009). Although floral development proceeds normally in *svp* and *agl24* mutants, *svp agl24* double mutants show floral abnormalities that increase in severity with increases in temperature (Gregis *et al.*, 2006). Overexpression of *AtSVP* causes aberrant floral morphology in transgenic *Arabidopsis* such as the formation of secondary flowers, sepaloid petals, and flowers with shoot-like structures (Masiero *et al.*, 2004; Liu *et al.*, 2007).

While the role of *SVP* genes in flowering time and floral primordia development appear to be conserved in some plants, other *SVP* genes can have rather diverse functions. The *SVP-like* genes from *Brassica campestris* (*BcSVP*) (Lee *et al.*, 2007*a*), *Eucalyptus grandis* (*EgrSVP*) (Brill and Watson, 2004), and *Poncirus trifoliate* (*PtSVP*) (Li *et al.*, 2010) are implicated both in floral transition and meristem identity based on their expression pattern and the overexpression phenotype in *Arabidopsis*. In *Antirrhinum majus* (snapdragon), the *SVP-like* gene *AmINCOMPOSITA* (*INCO*) has developed a more prominent role in floral meristem identity (Masiero *et al.*, 2004). *inco* mutants have no flowering-time phenotype, but display abnormal floral morphology that includes the lack of repression of prophyll development. In monocots, the three *SVP-like* genes in *Hordeum vulgare* (barley): *BARLEY MADS1 (HvBM1)*, *VEGETATIVE TO REPRODUCTIVE TRANSITION GENE 2* (*HvVRT2*), and *HvBM10 (HvVRT2-like)* are expressed in vegetative tissues. RNA interference (RNAi) constructs targeting either *BM1* or *BM10* did not result in any phenotypic abnormalities or change in heading time, but ectopic expression caused floral reversion in transgenic barley plants (Trevaskis *et al.*, 2007*b*). In *Oryza sativa* (rice), there are three *SVP-like* genes: *OsMADS22, OsMADS47*, and *OsMADS55.* Knock-down and over-expression lines showed that these genes appear to function in brassinosteroid signalling and shoot development (Duan *et al.*, 2006; Lee *et al.*, 2008). Heterologous expression of *OsMADS22* and *OsMADS47* in *Arabidopsis* caused floral defects (Fornara *et al.*, 2008) while that of *OsMADS55* led to delayed flowering (Lee *et al.*, 2012). In perennials, there is a role for SVP-like proteins in dormancy. Deletion of the six *SVP-like* genes [*DORMANCY-ASSOCIATED MADS-BOX 1–6* (*DAM1–6*) in *Prunus persica* (peach) resulted in failure to enter dormancy under cold or short-day induction (Bielenberg *et al.*, 2008; Jimenez *et al.*, 2009). In *Actinidia chinensis* (kiwifruit), a perennial vine, all of the four *SVP-like* genes (*AcSVP1–4*) were implicated in bud dormancy and their ectopic expression in *Arabidopsis* caused floral abnormalities. However, only *AcSVP1* and *AcSVP3* were able to complement the *Arabidopsis svp* mutant (Wu *et al.*, 2012).

*SVP-like* sequences were identified in another large and agronomically important plant family, the Fabaceae, but none have been functionally characterized (Hecht *et al.*, 2005; Jung *et al.*, 2012). Thus, the aim of this study was to investigate the role of *SVP-like* genes in regulating flowering time in the model legume, *Medicago truncatula* (*Medicago*). *Medicago* offers several advantages including a diploid and relatively modest genome of ~525Mb that has been sequenced, large collections of accessions with variation in flowering time, and extensive mutant populations for forward and reverse genetic screens (Julier *et al.*, 2007; Rose, 2008; Tadege *et al.*, 2008; He *et al.*, 2009; Branca *et al.*, 2011; Young *et al.*, 2011). Similar to the *Arabidopsis* winter annual accessions and winter varieties of wheat and barley, flowering in *Medicago* is promoted by a long day (LD)-photoperiod and vernalization and *Medicago FTa1*, an orthologue of *FT*, plays an important role in promoting flowering under these inductive conditions (Laurie *et al.*, 2011; Yeoh *et al.*, 2011). However, *Medicago* lacks flowering-time repressors that belong to the FLC-MADS AFFECTING FLOWERING (MAF) clade in the Brassicaceae family or genes that are similar to the VERNALIZATION2 (VRN2) flowering time repressor from cereals that are directly or indirectly repressed by vernalization and other pathways such as the *Arabidopsis* autonomous pathway (Hecht *et al.*, 2005; Trevaskis *et al.*, 2007*a*; Putterill *et al.*, 2013; Ruelens *et al.*, 2013).

Here, two *Medicago SVP-like* genes were identified and characterized by analysing their gene-expression patterns and by manipulating their expression in transgenic *Arabidopsis* and *Medicago* plants.

## Materials and methods

### Bioinformatics

The two *Medicago* (*Medicago truncatula*) *SVP-like* genes were identified by BLAST searches against the *Medicago* EST and BAC sequences. Alignment was performed using ClustalW in the Geneious software package [version 6.0.5 available from www.geneious.com (last accessed 1 November 2013) (Biomatters, Ltd.)]. A phylogenetic tree was generated using the Neighbor–Joining method via bootstrap resampling in the Geneious program. The accession numbers for the sequences used are as follows: *Medicago* MtSVP1 (Medtr5g032520), MtSVP2 (Medtr5g032150), Medtr5g066180; *Arabidopsis* AtSVP (At2G22540), AtAP1 (At1g69120), AtSOC1 (At2g45660), AtAGL14 (At4g11880), AtAGL19 (At4g22950), AtAGL24 (At4g24540); rice OsMADS22 (AB107957), OsMADS47 (AY345221), OsMADS55 (AY345223); barley HvBM10 (EF043040), HvVRT2 (DQ201168), HvBM1 (AJ249142); *Lolium perenne* (ryegrass) LpMADS10 (AAZ17549); *Euphorbia esula* (leafy spurge) EeDAM2 (ABY60423); peach PpDAM1 (DQ863253), PpDAM2 (DQ863255), PpDAM3 (DQ863256), PpDAM4 (DQ863250), PpDAM5 (DQ863251), PpDAM6 (DQ863252); *Brassica campestris* (Chinese cabbage) BcSVP (DQ922945); snapdragon AmINCOMPOSITA (CAG27846); *Eucalyptus grandis* EgrSVP (AY263809); *Populus trichocarpa* (poplar) PtSVP (XM_002310274); *Lotus japonicus* LjSVP (TC40194); *Pisum sativum* (garden pea) PsSVP (AY830919); *Glycine max* (soybean) GmSVP1 (Glyma01g02880), GmSVP2 (Glyma02g04710), GmSVP3 (Glyma06g10020), GmSVP4 (Glyma07g30040), GmSVP5 (Glyma08g07260), GmSVP6 (Glyma13g33040), GmSVP7 (Glyma15g06300), GmSVP8 (Glyma15g06310); kiwifruit AcSVP1 (JF838216), AcSVP2 (JF838217), AcSVP3 (JF838218), AcSVP4 (JF838219); and *Phaseolus vulgaris* (common bean) PvSVP (TC35086). These sequences were obtained from previous studies (Bielenberg *et al.*, 2008; Fornara *et al.*, 2008; Jung *et al.*, 2012; Wu *et al.*, 2012) and NCBI.

### Plant material and growth conditions

Jester and R108_C3 (R108) are the two *Medicago* genotypes used in this study. They belong to the two subspecies of *Medicago truncatula* Gaertn (barrel medic), ssp. *truncatula* and ssp. *tricycla*, respectively. The reverse genetic lines for *MtSVP2* were obtained from the Samuel Roberts Noble Foundation (Ardmore, Oklahoma). Scarification, germination, seed vernalization prior to growth in LD conditions (16/8h light/dark), and cultivation of *Medicago* plants were done as described previously (Laurie *et al.*, 2011; Yeoh *et al.*, 2013). For vernalization of seedlings, seeds germinated for 3 d at 21 °C were grown on soil in LD until a monofoliate and the first trifoliate leaf emerged (2-leaf stage of development, ~11 d in LD). Plants at this stage were vernalized at 4 °C for 2 weeks in controlled growth cabinets under short-day (SD) photoperiod (8/16h light/dark) with 40% humidity and then returned in LD. Flowering time of *Medicago* plants was measured in terms of the number of days from germination to flowering and/or the total number of nodes accumulated on the main axis at flowering.

The *Arabidopsis* wild type (WT) Columbia (Col) and *svp-41* mutant in the Col background were grown in LD. The *svp-41* mutant harbours a deletion of bases 193 and 194 in the *AtSVP* coding sequence (Hartmann *et al.*, 2000). The resulting frameshift creates a premature stop codon at the 69th amino acid (aa), generating a truncated 69 aa protein compared with the wild-type AtSVP protein of 240 aa in length. This mutation causes the *svp-41* mutant to flower earlier than WT Col plants.

### RNA extraction and quantitative reverse transcriptase-PCR (qRT-PCR) analysis

RNA extraction, cDNA synthesis, and qRT-PCR were performed as previously described (Laurie *et al.*, 2011; Yeoh *et al.*, 2013). The identity of the PCR amplicons was checked by DNA sequencing. For gene expression analyses, leaf or shoot apical buds were harvested from three-leaf (fully-expanded) stage plants, unless otherwise stated. In the developmental time-course, flower buds were small unopened flowers, while flowers were open flowers. The data were normalized to the housekeeping gene, *PROTODERMAL FACTOR 2* (*PDF2*) or *TUBULIN*. The primers used are shown in Supplementary Table S1 at *JXB* online.

### Generation of transgenic *Arabidopsis* plants

**Fig. 1.**
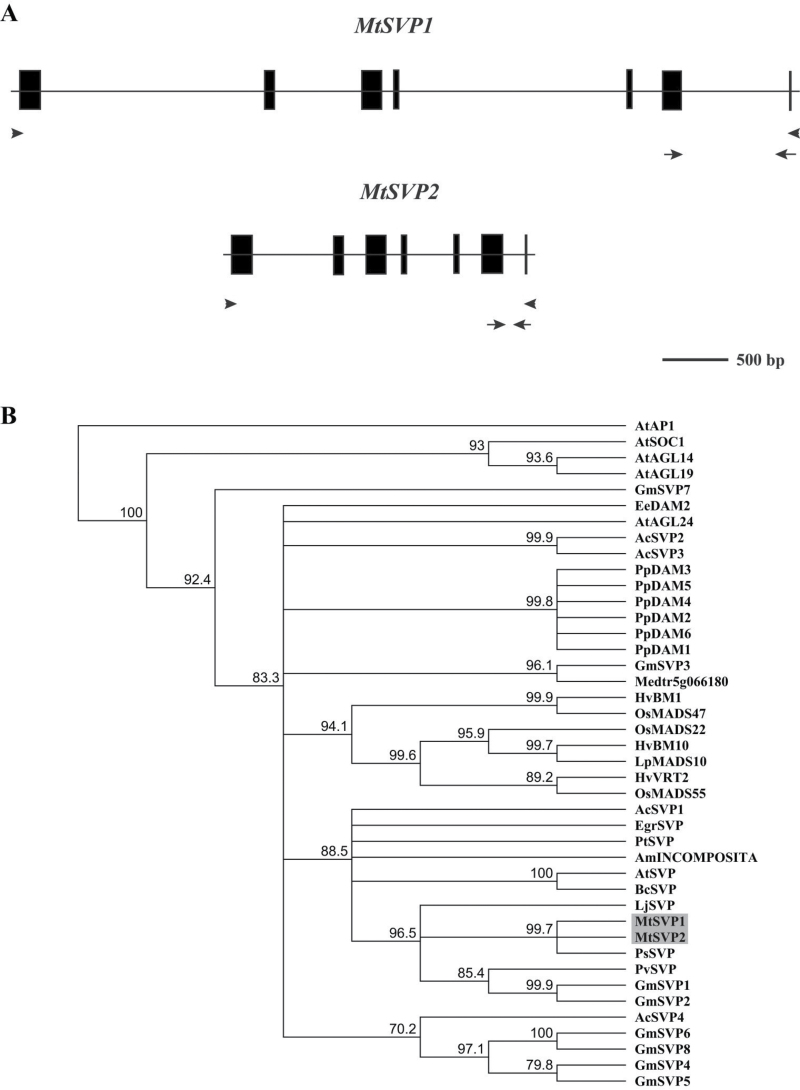
Genomic structures of *MtSVP1* and *MtSVP2* and phylogenetic analysis of SVP-like proteins from different species. (A) The exons are indicated by black boxes and introns by a thin line. The arrows indicate the positions of primers used for quantitative real-time PCR while the arrowheads represent the primers used for cloning the full-length *MtSVP1* and *MtSVP2* coding sequences from *Medicago* cDNA. (B) A consensus phylogenetic tree based on the amino acid alignment of MtSVP1 and MtSVP2 predicted proteins (shaded box) and SVP-like proteins from other species. The tree was generated using the Neighbor–Joining (NJ) method via bootstrap resampling with support threshold of 55% and rooted on AtAP1. The numbers indicate the bootstrap values based on 1000 replicates. Ac, *Actinidia chinensis*; Am, *Antirrhinum majus*; At, *Arabidopsis thaliana*; Bc, *Brassica campestris*; Ee, *Euphorbia esula*; Egr, *Eucalyptus grandis*; Gm, *Glycine max*; Hv, *Hordeum vulgare*; Lj, *Lotus japonicas*; Lp, *Lolium perenne*; Mt, *Medicago truncatula*; Os, *Oryza sativa*; Pp, *Prunus persica*; Ps, *Pisum sativum*; Pt, *Poncirus trifoliate*; and Pv, *Phaseolus vulgaris*. (This figure is available in colour at *JXB* online.)

In order to generate *MtSVP* overexpression (*35S:MtSVP*) constructs, the cDNAs of *MtSVP1* and *MtSVP2* were amplified using the Phusion Hot Start High-Fidelity DNA Polymerase (New England Biolabs) with gene-specific primers (Fig. 1A; see Supplementary Table S1 at *JXB* online) and then cloned in pCR8/GW/TOPO TA (TOPO) entry vector (Invitrogen, Corporation, USA). The entry clones were linearized with 5U *Xho*I and then recombined into the plant transformation vector, pB2GW7 (Karimi *et al.*, 2002) to create expression clones using the GATEWAY TECHNOLOGY kit (Invitrogen, Corporation, USA) according to the manufacturer’s instructions. Entry and expression clones were subjected to restriction enzyme digestion and sequencing to check for the correct orientation and the absence of mutation in the constructs. Clones were transformed by heat shock into One Shot TOP10 Chemically Competent *E. coli* (Invitrogen, Corporation, USA) according to the manufacturer’s instructions. *Agrobacterium tumefaciens* strain GV3101 with expression clones was applied to *Arabidopsis* WT Col and *svp-41* mutant flower buds as previously described by Martinez-Trujillo *et al.*, (2004). Seeds were directly sown onto soil and transformants were selected using the Basta herbicide (10mg l^–1^).

### Transformation and regeneration of *Medicago* R108

Transformation of *Medicago* R108 and regeneration via somatic embryogenesis was conducted as previously described by Laurie *et al.* (2011) with minor modifications. Two rounds of transformation were conducted with *35S:MtSVP1* or vector only (pB2GW7) as a control. The control group went through somatic embryogenesis on selective or non-selective medium as the negative control or the regeneration control, respectively. The transformant plants were selected with 1–2mg l^–1^ of dl-phosphinothricin (Sigma). Regenerated T_0_ transformed plantlets were transferred to soil and sprayed again with 120mg l^–1^ Basta (Kiwicare) to confirm their transgenic nature. PCR-genotyping also confirmed the presence of the transgenes.

### Reverse genetic screening for *Tnt1* insertions and linkage analysis

Mutant plants with *Tnt1* insertions at the *MtSVP* genes were screened using a reverse genetic approach (Tadege *et al.*, 2008). *Tnt1* insertions were identified in the Noble Foundation, USA using *Tnt1*- and gene-specific primers. No lines were found to have *Tnt1* insertion at the *MtSVP1* locus while two different lines have *Tnt1* sequences inserted within the *MtSVP2* gene, one within intron 4 in line NF11029 and one within intron 6 in line NF13617. The lines were grown under LD and vernalized LD (VLD) conditions. Their gDNA was extracted and the nature and direction of *Tnt1* insertion in the lines were confirmed using PCR. The expression levels of *MtSVP2* were measured in the transgenic homozygous plants from the two lines using qRT-PCR. All the primers used are shown in Supplementary Table S1 at *JXB* online.

## Results

### Identification of two closely related *SVP-like* genes in *Medicago*

The top-scoring hits from interrogation of *Medicago* genomic and EST sequences using BLAST analyses with the AtSVP protein were two closely-related *SVP-like* genes named *MtSVP1* and *MtSVP2*. *MtSVP1* (Medtr5g032520) (Hecht *et al.*, 2005) and *MtSVP2* (Medtr5g032150) encode MADS-domain proteins with 89% aa identity to each other and 70% and 66% aa identity respectively, with the AtSVP protein. MtSVP1 and MtSVP2 were less similar to AGL24, which is the most closely related MADS domain protein to SVP (Parenicova *et al.*, 2003), with 56% and 55% aa identity, respectively. Alignment with SVP/AGL24-like proteins indicates that the MADS-box, intervening (I-box) region, and keratin-like (K-box) motif are well conserved in MtSVP1 and MtSVP2 (see Supplementary Fig. S1 at *JXB* online) consistent with the features shared by the MADS-domain family of transcription factors in plants (Parenicova *et al.*, 2003). *MtSVP1* and *MtSVP2* have a comparable genomic structure with 7 exons, but *MtSVP1* is a bigger gene due to having larger introns (Fig. 1A).

In a Neighbor–Joining phylogenetic tree, MtSVP1 and MtSVP2 grouped with eudicot SVP/AGL24 proteins in a clade that is distinct from other *Arabidopsis* MADS-box proteins such as SOC1, AGL14 and AGL19 (Fig. 1B). The two MtSVP proteins and other legume SVP proteins formed a sister clade to AtSVP with MtSVP1 and MtSVP2 being most closely-related to pea PsSVP. The monocot SVP-like proteins from rice (OsMADS47, OsMADS55, and OsMADS22), barley (HvBM1, HvBM10, and HvVRT2) and ryegrass (LpMADS10) formed an independent sub-clade, distinct from the eudicot group.

A third candidate *MtSVP/AGL24* gene, Medtr5g066180 (Hecht *et al.*, 2005) encoded a protein that was less similar to AtSVP (48% aa identity) than were MtSVP1 and MtSVP2. The divergence of Medtr5g066180 compared with MtSVP1 and MtSVP2 was also observed in the phylogenetic tree as it fell outside the clade containing AtSVP, MtSVP1, and MtSVP2 (Fig. 1B). It was slightly more similar to AGL24 (54% aa identity) but did not fall into an AGL24 clade. Thus, further work focused on investigating the expression and function of *MtSVP1* and *MtSVP2*.

### Gene expression patterns of *MtSVP1* and *MtSVP2*

**Fig. 2.**
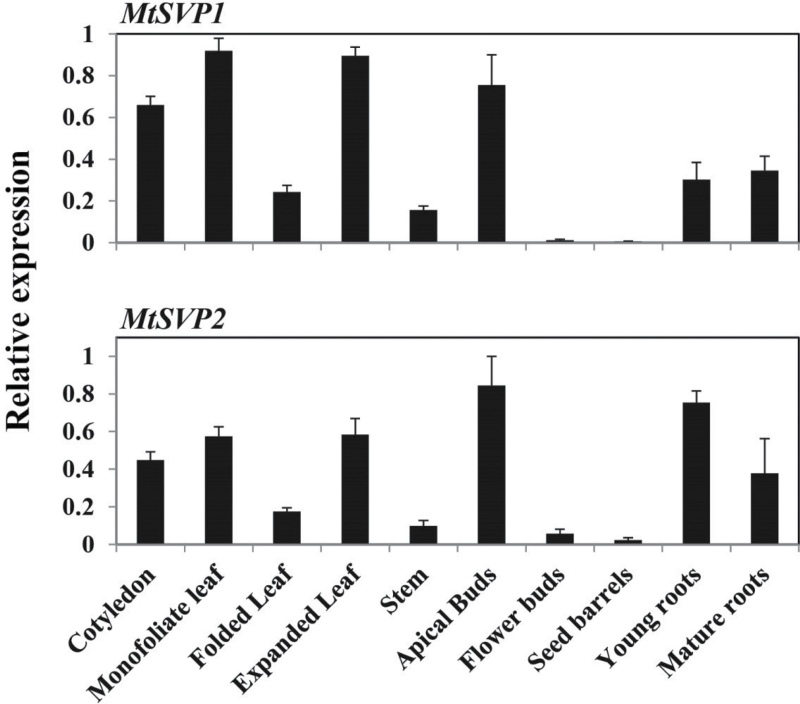
Expression of *MtSVP1* and *MtSVP2* in different tissues of *Medicago* wild-type Jester plants. Plants were grown in LD after 2 weeks of vernalization at 4 °C in the dark. The young roots and cotyledons were from 5-d-old seedlings; monofoliate leaf, folded and expanded trifoliate leaves, stem, apical buds, and mature roots from 15-d-old plants; flower buds (young) from 23-d-old flowering plants; and seed barrels from 35-d-old plants. Gene expression was determined using qRT-PCR and the data are shown as the mean ±standard error (SE) of three biological replicates, which were normalized to *Medicago PROTODERMAL FACTOR2* (*PDF2*). The data are presented relative to the highest value. Tissues were harvested at 2h after dawn [Zeitgeber time (ZT) 2]. (This figure is available in colour at *JXB* online.)

To gain insight into the possible roles of *MtSVP1* and *MtSVP2* in plant development, their gene expression profiles were analysed using qRT-PCR. First, transcript abundance in different tissues of vernalized long-day grown (VLD) *Medicago* wild type (WT) plants was compared (Fig. 2). Both *MtSVP1* and *MtSVP2* were expressed during vegetative development in all the tissues tested; most highly in cotyledons, monofoliate leaves, expanded trifoliate leaves, and apical buds. Transcripts of both genes were also present in folded leaves, stems, and young and mature roots and were detected at much lower levels in young flower buds and developing seed barrels.

**Fig. 3.**
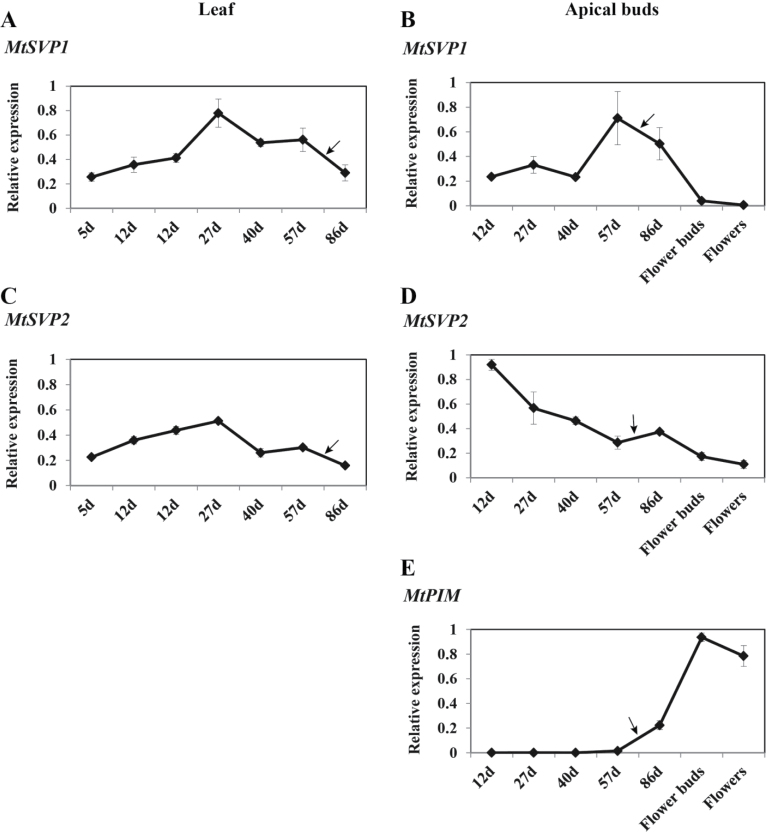
Developmental regulation of *MtSVP1* and *MtSVP2* in leaves and shoot apices of *Medicago* wild-type R108 plants. Relative expression levels of *MtSVP1* and *MtSVP2* in fully-expanded trifoliate leaves (A, C) and uppermost apical buds (B, D) harvested from plants of increasing developmental ages grown in LD. The arrows indicate the time of flowering. The floral meristem identity gene, *PROLIFERATING INFLORESCENCE MERISTEM* (*MtPIM)* was used as a molecular marker of the floral transition (E). Tissues were harvested at ZT2. Gene expression was determined using qRT-PCR and the data are shown as the mean ±standard error (SE) of three biological replicates, which were normalized to *PDF2*. The data are presented relative to the highest value in both tissues for each gene. (This figure is available in colour at *JXB* online.)

The expression of *MtSVP1* and *MtSVP2* in leaves and shoot apices was examined in more detail over a developmental time-course in *Medicago* WT R108 plants in LD conditions (Fig. 3). The expression of the *AP1-like* gene, *PROLIFERATING INFLORESCENCE MERISTEM (PIM1*) was used as a marker of the transition to flowering (Fig 3E) together with the appearance of the first visible floral buds. Consistent with the results shown in Fig. 2, both genes were expressed throughout development in leaves and shoot apices, with expression declining to its lowest level in mature flowers. In leaves, *MtSVP1* gene expression increased 3–4-fold after 27 d of growth and then gradually declined back to the levels observed in young vegetative plants, while *MtSVP2* gene expression was weakly elevated by about 2-fold at 27 d before decreasing back to the levels seen in young plants (Fig. 3A, C).

More marked differences in gene expression were observed between the two genes in their developmental pattern of expression in apical buds. *MtSVP1* transcript was abundant in apical buds from the early stages of vegetative development, up-regulated further by around 3-fold just prior to the floral transition, and then gradually decreased after the onset of flowering with a strong decline in open flowers (Fig. 3B). *MtSVP2* was highly expressed in apical buds in young vegetative plants and transcript levels declined throughout development with the lowest level detected in mature flowers (Fig. 3D). While at lower levels relative to other developmental stages, transcripts of both *MtSVP* genes were readily detectable in flower buds and open flowers. The effect of extended cold temperatures experienced by seedlings during vernalization treatment on the expression levels of the *MtSVP* genes was also investigated. However, no direct effects of cold temperatures on the transcript abundance of either of the *MtSVP* genes in leaves and apical buds were seen (see Supplementary Fig. S2 at *JXB* online).

### Overexpression of the two *Medicago SVP-like* genes in *Arabidopsis* delays flowering and causes floral defects

To assess the potential roles of the two *MtSVP* genes in flowering-time control and the regulation of flower development, they were overexpressed in the heterologous plant *Arabidopsis*. cDNA clones of the *MtSVP* genes were generated from total RNA of Jester wild-type plants, fused to the cauliflower mosaic virus (CaMV) 35S promoter (*35S:MtSVP1* and *35S:MtSVP2*) and introduced into *Arabidopsis* WT Col plants. Nine T_1_ *35S:MtSVP1* and *35S:MtSVP2* plants with single locus insertions were identified (see Supplementary Table S2A at *JXB* online).

**Fig. 4.**
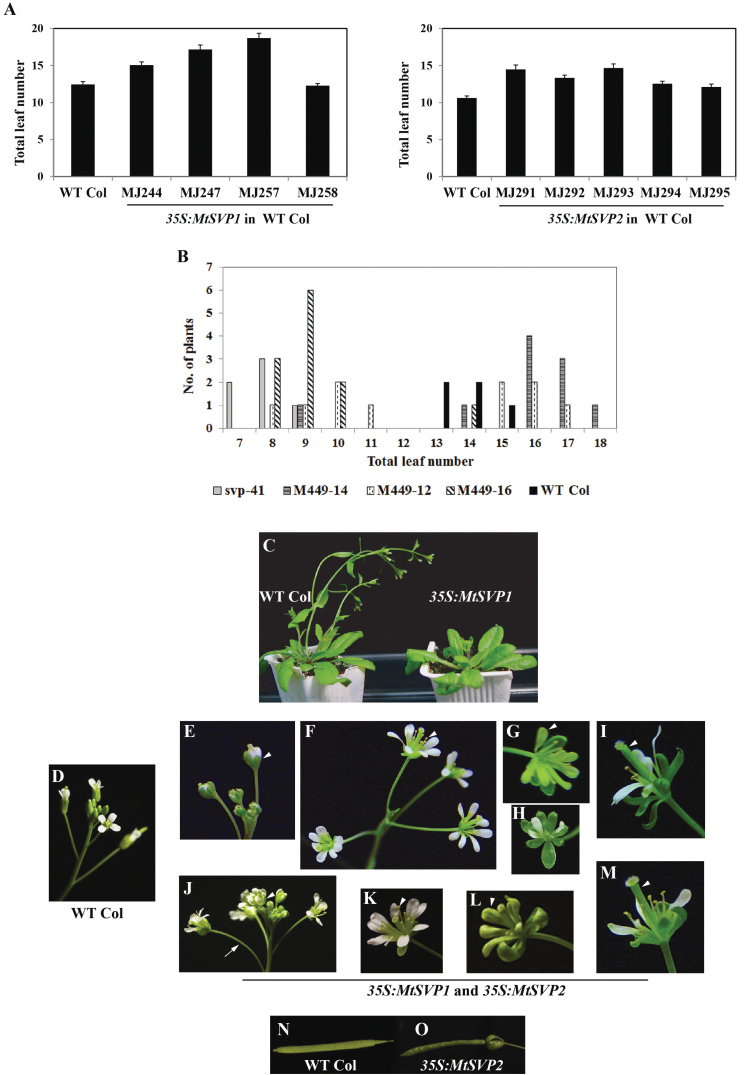
Flowering time and floral phenotypes of transgenic *Arabidopsis* plants overexpressing *MtSVP1* and *MtSVP2*. (A) Graphs showing the effect of ectopic expression of *MtSVP1* (left) and *MtSVP2* (right) on the flowering time of *Arabidopsis* WT Col. The flowering time of transgenic T_2_ plants from four independent *35S:MtSVP1* Col lines (*n*=17–19) and five independent *35S:MtSVP2* Col lines (*n*=15–18) is shown. Col WT plants were grown as a control (*n*=9–24). Plants were grown in LD until they flowered. Flowering time was measured as the total number of rosette and cauline leaves at flowering and is shown as the mean ±SE. The flowering time of the *35S:MtSVP* lines was significantly different from WT (*P* <0.01; *t*-test) except for MJ258. (B) Overexpression of *MtSVP2* complements the *Arabidopsis svp-41* mutation. Histograms showing the flowering time measured as the total number of rosette and cauline leaves at flowering of *svp-41* mutants (n=6), WT Col (*n*=5), and three segregating T_2_ populations from the overexpression of *MtSVP2* in the *Arabidopsis svp-41* background (*n*=10–12). (C) Photograph showing the delayed flowering phenotype of transgenic *Arabidopsis* plants overexpressing *MtSVP1* (right) compared with WT Col of the same age. (D–O) Photographs of inflorescence, flowers, and siliques of WT Col (D, N) and transgenic plants from *35S:MtSVP1* (E–I) and *35S:MtSVP2* (J–M, O) Col lines. Plants overexpressing the *MtSVP* genes displayed variable floral abnormalities that include failure of sepals to enclose the floral bud at the early stages of development (arrowheads in E and J); irregular number of petals (F, K), sepals (H), and anthers (arrowheads in F and K); splayed arrangement of floral organs (I, M); enlarged carpels (arrowheads in I and M); sepaloid (green) petals (arrowheads in G and L); longer pedicel than WT (arrow in J); and lack of sepal abscission in wrinkled, narrow siliques (O).

The T_2_ progeny of eight of these transgenic lines had delayed flowering compared with WT Col plants (Fig. 4A, C) as well as floral defects (Fig. 4D–O), with the severity of these correlating with the delay in flowering time (see Supplementary Table S2 at *JXB* online). At the onset of flower formation, the transgenic plants were easily distinguished from WT Col (Fig. 4D) by the appearance of enlarged sepals surrounding a more splayed arrangement of floral organs (Fig. 4E, J). At later stages, other defects became more apparent and the most extreme ones were characterized by irregular symmetry of floral organ arrangement, alterations in petal and anther number, elongated carpels, deformed siliques, sepalloid (green) petals and long pedicels (Fig. 4F–I, K–M). In some cases, the sepals remained larger and much greener than the wild type and did not abscise even when siliques had developed (Fig. 4H, L, O).

*MtSVP2* was also overexpressed in the *Arabidopsis* early flowering mutant, *svp-41*. Two out of three independent transgenic lines showed rescue of the flowering-time phenotype in the T_2_ generation (Fig. 4B). As observed for *35S:MtSVP* plants in the Col background, some *MtSVP2 svp-41* T_2_ plants flowered later than WT Col plants (Fig. 4B) and had floral abnormalities (data not shown).

### Overexpression of *MtSVP1* in *Medicago* results in longer pedicel length and other changes in flower development

**Fig. 5.**
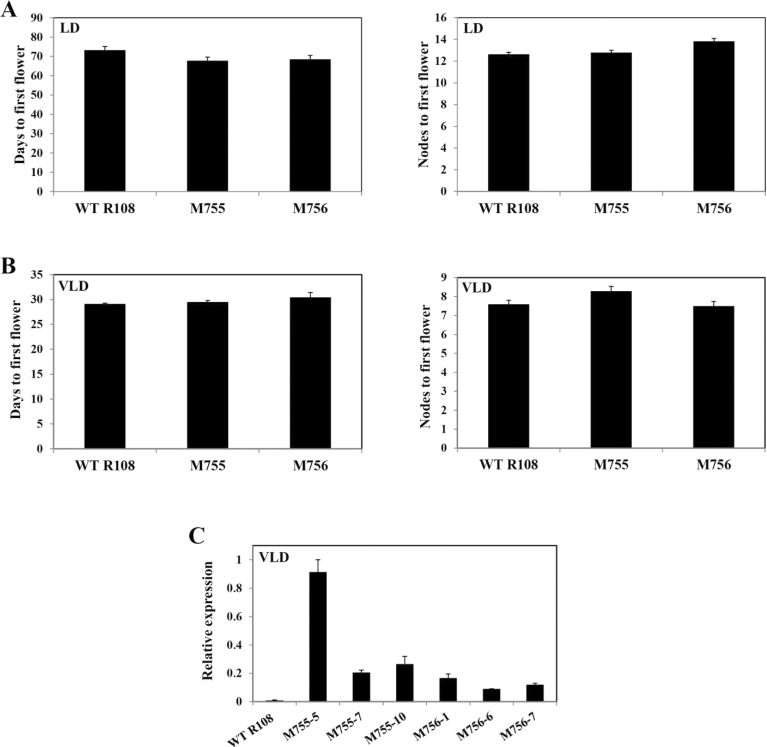
Overexpression of *MtSVP1* in *Medicago* has no effect on flowering time. (A) Flowering time in days to first flower and nodes to first flower of WT R108 (*n*=8) and transgenic T_1_ *Medicago* plants from two independent *35S:MtSVP1* R108 lines (*n*=11–14) grown in LD. (B) Flowering time in days to first flower and nodes to first flower of WT R108 (*n*=8) and transgenic *35S:MtSVP1* T_1_ plants from two independent lines (*n*=7–10) grown in LD conditions after 14 d of vernalization (VLD). The flowering time in (A) and (B) is shown as the mean ±SE. (C) *MtSVP1* transcript accumulation in fully expanded trifoliate leaves of WT R108 and six *35S:MtSVP1* T_1_ plants from two independent T_0_ transformants grown in VLD. Gene expression was determined using qRT-PCR and the data are shown as the mean ±SE of two biological replicates, which were normalized to *TUBULIN*. The data are presented relative to the highest value. The leaf tissues were harvested at ZT2. (This figure is available in colour at *JXB* online.)

Next, the effect of ectopic *MtSVP1* expression on *Medicago* flowering time and floral development was investigated. WT R108 leaflets were transformed by co-cultivation with *Agrobacterium tumefaciens* containing the *35S:MtSVP1* construct and plants were regenerated from three different leaflets via somatic embryogenesis (Cosson *et al.*, 2006). The average flowering time of the T_1_ progeny from two independent T_0_ regenerated *35S:MtSVP1* lines was similar to the WT R108 plants grown in LD and VLD conditions (Fig. 5A, B) The T1 *35S:MtSVP1* plants flowered at the same time as WT R108 despite elevated expression of *MtSVP1* in leaves (Fig. 5C).

**Fig. 6.**
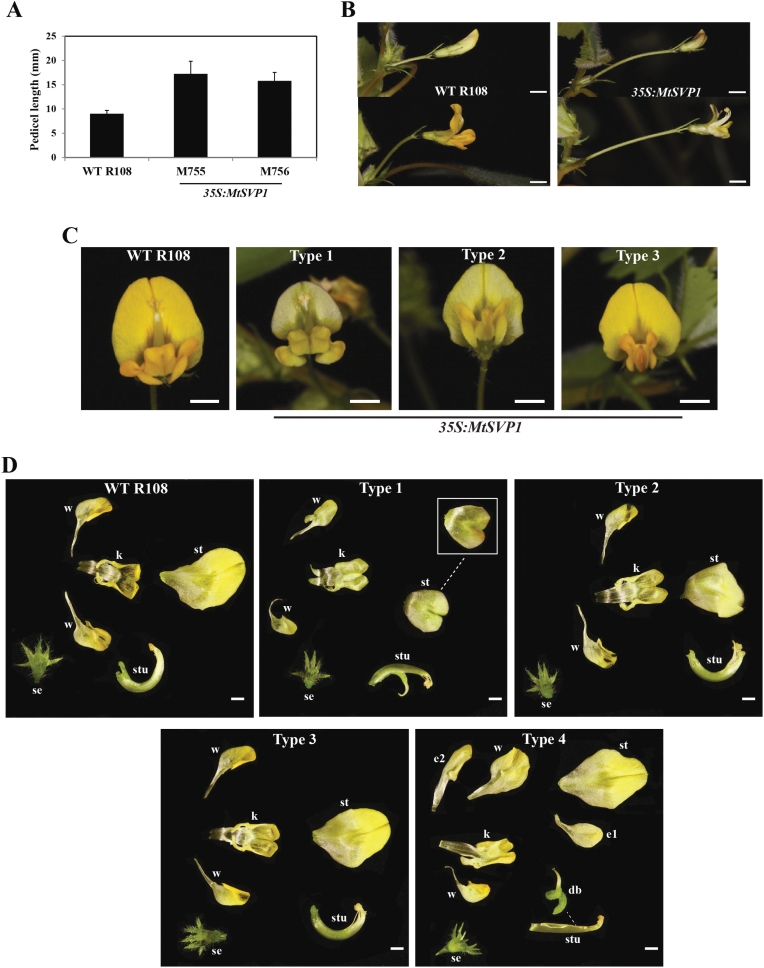
Overexpression of *MtSVP1* in *Medicago* results in longer pedicels and smaller and abnormal flowers. (A) Average pedicel length of WT R108 and *35S:MtSVP1* T_1_ transformants from two lines, M755 and M756. The data are shown as the mean ±SE of 51 flowers from WT R108 (*n*=5 plants), 54 flowers from M755 (*n*=5 plants), and 52 flowers from M756 (*n*=7 plants). The plants were grown in LD after 14 d of cold treatment. The pedicel length was measured from the base of a pedicel to the base of the attached open flower. (B) Photographs showing flowers of WT R108 and transgenic *35S:MtSVP1* plants at the same magnification. (C, D) Flower phenotypes of WT R108 and transgenic *Medicago 35S:MtSVP1* T_1_ plants. Five types of flowers were observed in *35S:MtSVP1* transformants compared with WT R108. Flowers were either similar to WT R108 or had abnormalities (Type 1–Type 4). Type 1 flowers were the smallest flowers observed and were paler in colour compared with WT R108. There was also a purple patch at the back side of standard petal, which appeared to be in a more open form. Type 2 flowers were paler in colour, slightly bigger than Type 1 flowers but still smaller compared with WT R108. Type 3 flowers were the same colour as WT R108, slightly bigger than Type 1 flowers, but still smaller than WT R108. The photos are shown in the same scale. (D) Photographs of floral organs from WT R108 and the different types of flowers from *35S:MtSVP1* transformants as explained above. The inset in Type 1 is the back side of the standard petal showing the purple patch. Another type of flower with extra petals (e1 and e2) and sepal (se) was also seen amongst the *35S:MtSVP1* transformants (Type 4). St, standard petal; w, wing petal; k, keel petal; stu, staminal tube surrounding the carpel; db, developing seed barrel. The photos are shown in the same scale. Bars=2mm in (B), 1.29mm in (C), and 1mm in (D).

Some changes to floral development were observed in the transgenic lines. Flowers of T_1_ plants from both *35S:MtSVP1* lines had significantly longer pedicels than WT plants (Fig. 6A, B). In addition, some of the flowers from the *MtSVP1* plants were smaller than WT flowers with altered petal shape and colour (Fig. 6C, D) and, in a single case, a flower with extra petals and sepals was observed amongst the *35S:MtSVP1* transformants (Fig. 6D).

### Analysis of plants carrying *Tnt1* retroelement insertions in *MtSVP2*

**Fig. 7.**
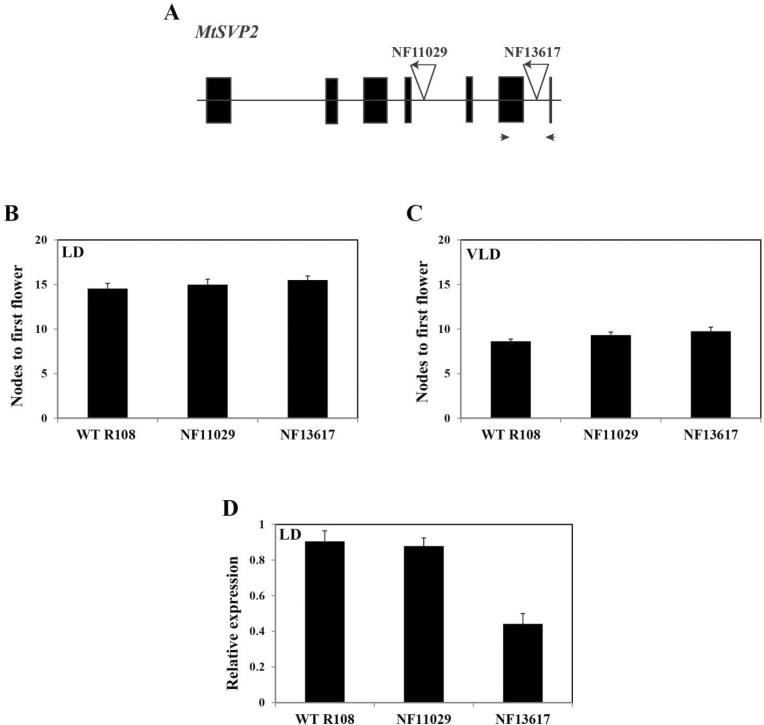
Flowering time and gene expression of *Medicago* R108 plants with *Tnt1* insertions at *MtSVP2*. (A) Schematic diagram of the *MtSVP2* gene with the positions of *Tnt1* insertions in the two lines, NF11029 (*Tnt1* inserted within intron 4) and NF13617 (intron 6). Exons are shown as black boxes and introns as thin lines. The arrowheads indicate the positions of primers used for qRT-PCR. (B) Flowering time of WT R108 (*n*=11) and transgenic plants with homozygous *Tnt1* insertion at *MtSVP2* in lines NF11029 (*n*=8) and NF13617 (*n*=14) under LD conditions. (C) Flowering time of WT R108 (*n*=11) and transgenic homozygous plants from lines NF11029 (*n*=3) and NF13617 (*n*=4) under vernalized LD (VLD) conditions. The flowering time in (B) and (C) was measured in nodes to first flower and is shown as the mean ±SE. (D) Relative expression levels of *MtSVP2* in fully expanded trifoliate leaves of WT R108 and homozygous plants from lines NF11029 and NF13617 grown under LD conditions (46-d-old). Gene expression was determined using qRT-PCR and the data are shown as the mean ±SE of three biological replicates, which were normalized to *PDF2*. The data are presented relative to the highest value. Tissues were harvested at ZT2. (This figure is available in colour at *JXB* online.)

As part of the effort to elucidate the role of the *MtSVP* genes in *Medicago* flowering, a reverse genetic approach was used on a population of R108 *Tnt1* retrotransposon-tagged mutants (Tadege *et al.*, 2008) with the aim of finding plants carrying insertion mutations in the two *MtSVP* genes. No lines were found with a *Tnt1* insertion at the *MtSVP1* locus. Two lines were identified with *Tnt1* insertions within the *MtSVP2* gene. Line NF11029 had an insertion in intron 4 and line NF13617 had one within intron 6 (Fig. 7A). *MtSVP2* expression was unaffected in NF11029, but partially knocked down in NF13617 (Fig. 7D). The flowering time (Fig. 7B, C) and flower development (data not shown) were unaffected in both *Tnt1* insertion lines.

## Discussion

In this study, two *Medicago SVP-like* genes, *MtSVP1* and *MtSVP2*, were identified whose predicted proteins clustered within the eudicot SVP clade separately from AGL24 and other MADS-domain transcription factors. They are more closely related to pea PsSVP than to other legume SVP-like sequences consistent with the high degree of synteny between pea and *Medicago* (Choi *et al.*, 2004). The presence of two *SVP-like* genes in *Medicago,* as opposed to one gene in *Arabidopsis,* is likely to have resulted from the proposed whole genome duplication event in the papilionoid subfamily of the Fabaceae that occurred ~58 million years ago, after the divergence of the fabids and malvids (Bell *et al.*, 2010; Young *et al.*, 2011).

In *Medicago, MtSVP1* and *MtSVP2* are both highly expressed in vegetative tissues and are detected, but at much lower levels in young unopened flower buds and open flowers. This pattern has similarity to *AtSVP* and is consistent with a possible role of the *MtSVP* genes as flowering-time repressors during the vegetative phase. In addition, both *MtSVP* genes delayed flowering when ectopically expressed in *Arabidopsis* and *MtSVP2* rescued the early flowering phenotype of *svp-41* mutants, indicating that the two *Medicago SVP* genes have a similar function to *SVP* as repressors of flowering in the *Arabidopsis* heterologous system.

The *35S:MtSVP* transgenic *Arabidopsis* plants also had aberrant flowers with all the four whorls affected in number and/or morphology and pedicels that were longer than in the wild type. Some flowers displayed vegetative features including green petals and leafy sepals that did not abscise. These floral abnormalities resembled, but were not identical to the phenotypes seen when *AtSVP* or other *SVP-like* genes from other plant species were overexpressed in *Arabidopsis*. The increase in petal and anther number was similar to *35S:BcSVP* transgenic plants (Lee *et al.*, 2007*a*) while the leafy sepals and/or green petals were also seen in the overexpression phenotype of *AtSVP* (Masiero *et al.*, 2004), *LpMADS10* (Petersen *et al.*, 2006), *HvBM1, HvBM10* (Trevaskis *et al.*, 2007*b*), *OsMADS22, OsMADS47* (Fornara *et al.*, 2008), and *AcSVP3* (Wu *et al.*, 2012), among others. Thus, despite having similar effects on flowering time in transgenic *Arabidopsis*, these differences in floral phenotypes indicate that the *Arabidopsis* and *Medicago* SVP proteins do not have identical biochemical functions in *Arabidopsis*. The enhanced vegetative features of flowers in *MtSVP*-overexpressing *Arabidopsis* lines may be the result of disrupting floral meristem identity and ensuing development due to ectopic expression of *MtSVP* in the inflorescence meristem and/or prolonged expression of the *MtSVP* genes in flower buds.

The similar sequences, *Medicago* expression patterns, and effects on flowering time and floral development in *Arabidopsis* suggest that the two *MtSVP* genes may have overlapping functions in *Medicago*. Interestingly, in contrast to transgenic *Arabidopsis*, overexpression of *MtSVP1* did not alter flowering time in *Medicago*. Thus, it is possible that the absence of key SVP protein interaction partners in *Medicago* such as FLC means that the *Medicago SVP* genes do not have a major role in regulating flowering time in WT *Medicago*. In addition, expression of the two *MtSVP* genes is not regulated by extended exposure to cold temperatures as is *FLC*, indicating that the *MtSVP* genes themselves are not directly targeted by the vernalization pathway in *Medicago*. On the other hand, some floral phenotypes were observed in the *35S*:*MtSVP1* transgenic *Medicago* plants. Floral defects included flowers with longer pedicels, as seen in transgenic *Arabidopsis* and, in some cases, novel phenotypes such as smaller flowers than WT with aberrant petal sizes, shapes, and colours. These results hint at a role for *MtSVP1* in *Medicago* flower development; a possibility given that the *MtSVP* genes are expressed in flowers at different stages of development. Alternatively, ectopic expression of *MtSVP1* may have interfered with the proper make-up of floral meristem and floral organ homeotic patterning complexes involving MADS-domain transcription factors (De Folter *et al.*, 2005; Lee *et al.*, 2007*a*; Fornara *et al.*, 2008).

As the two *MtSVP* genes are likely to be partially redundant, functional studies of the *MtSVP* genes could be developed further by analysing transgenic *Medicago* plants with knockdown of expression of both *MtSVP* genes. Another exciting approach would be to analyse MtSVP regulatory targets at a genome-wide level using techniques such as ChiP-sequencing (ChIP-seq), which have recently revealed that AtSVP directly regulates the expression of a large number of *Arabidopsis* genes implicated in many different plant processes (Tao *et al.*, 2012; Gregis *et al.*, 2013).

## Supplementary data

Supplementary data can be found at *JXB* online.

Supplementary Fig. S1. Multiple sequence alignment of SVP-like proteins from different plant species.

Supplementary Fig. S2. Effect of vernalization on the transcript levels of *MtSVP1* and *MtSVP2.*

Supplementary Table S1. List of oligonucleotide primers (5′–3′) used in this study.

Supplementary Table S2. Flowering time of T_2_ transgenic plants from the overexpression of *MtSVP1* and *MtSVP2* in WT Col under LD conditions and classification of floral phenotypes.

Supplementary Data
