# Supplementary material for: Overexpression of Medicago SVP genes causes floral defects and delayed flowering in Arabidopsis but only affects floral development in Medicago
Source: J Exp Bot. 2013 Nov 18;65(2):429–42. doi: 10.1093/jxb/ert384 (PMC3904704; doi:10.1093/jxb/ert384)
Supplement: Supplementary Data [file supp_65_2_429__index.html]

Over-expression of Medicago SVP genes causes floral defects and delayed flowering in Arabidopsis but only affects floral development in Medicago — Overexpression of Medicago SVP genes causes floral defects and delayed flowering in Arabidopsis but only affects floral development in Medicago — Supplementary Data 

# Overexpression of *Medicago SVP* genes causes floral defects and delayed flowering in *Arabidopsis* but only affects floral development in *Medicago*

## Supplementary Data

Data files

**Files in this Data Supplement:**

- Supplementary Data - Supplementary Data
