# Supplementary material for: Overexpression of Medicago SVP genes causes floral defects and delayed flowering in Arabidopsis but only affects floral development in Medicago
Source: J Exp Bot. 2013 Nov 18;65(2):429–42. doi: 10.1093/jxb/ert384 (PMC3904704; doi:10.1093/jxb/ert384)
Supplement: Supplementary Data [file supp_ert384_jexbot110742_file001.pdf]

Overexpression of Medicago *SVP* genes causes floral defects and delayed flowering in Arabidopsis but only affects floral development in Medicago

Mauren Jaudal, Jacob Monash, Lulu Zhang, Jiangqi Wen, Kirankumar S. Mysore, Richard Macknight and Joanna Putterill

Supplementary Data

Fig. S1. Multiple sequence alignment of SVP-like proteins from different plant species. The deduced amino acid sequences were aligned using ClustalW plugin available in the Geneious software package.

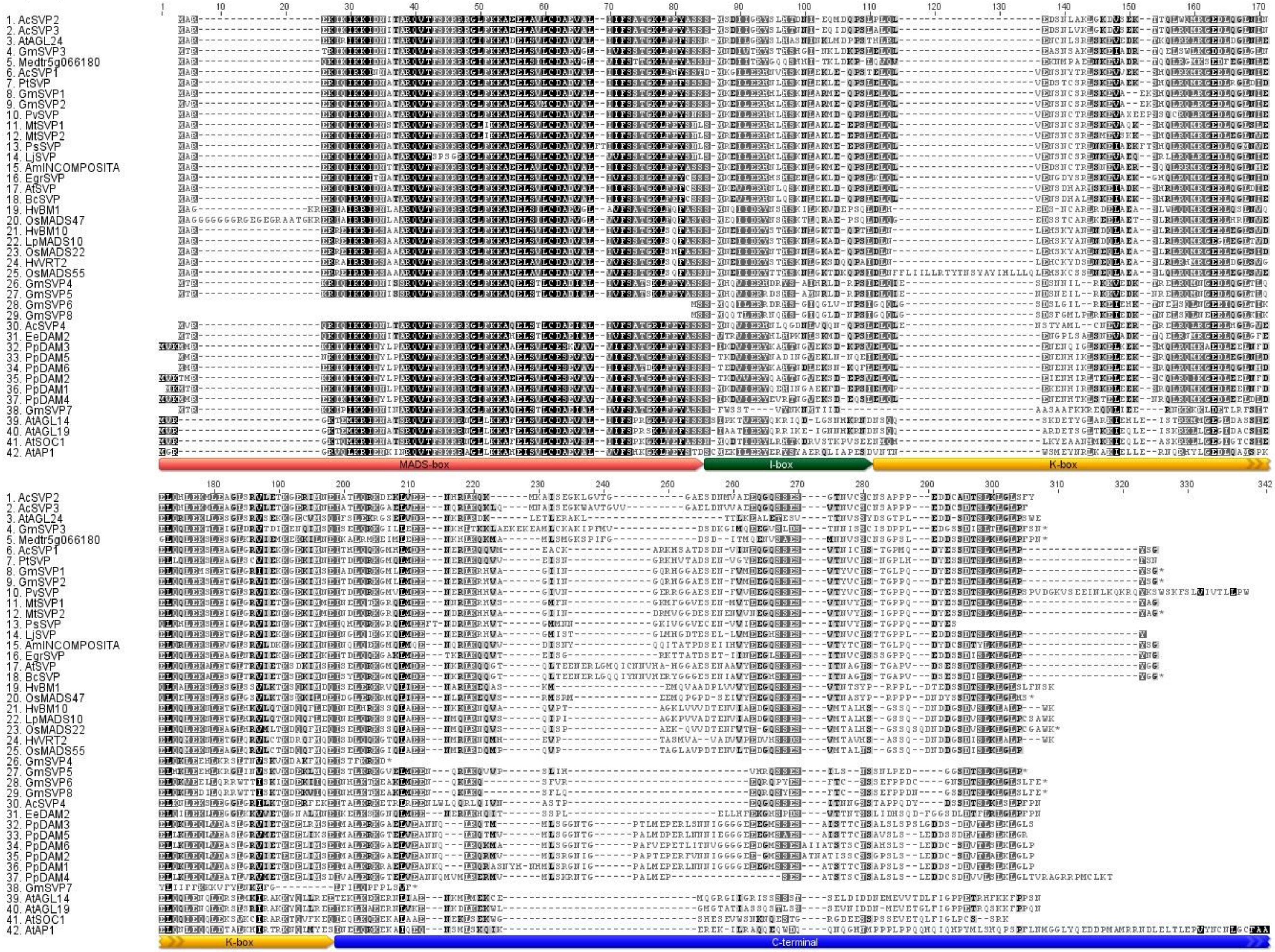

Fig. S2. Effect of vernalization on the transcript levels of *MtSVP1* and *MtSVP2.* (A) Schematic diagram of Medicago developmental stages prior to, during and after vernalization and the position of leaves that were sampled and used in gene expression analyses. Imbibed seeds of Medicago wild type Jester were grown in LD at 21 °C until 2-leaf stage in which the plants had a monofoliate (M) and fully expanded first trifoliate (L1) leaves (Time 0; approximately 11-day old). The plants were then vernalized for 14 days in SDs (16h dark/ 8h light) at 4 °C (14VT0=stands for 14 days in the cold, 0 day in the warm) and then returned to warm temperature (21 °C) under LD conditions for 2 (14VT2), 7 (14VT7), and 14 (14VT14) days. RNA samples were extracted from apical buds and leaves (encircled) at each stage. L1= Leaf 1, L3= Leaf 3, L6= Leaf 6. (B-C) Relative expression levels of *MtSVP1* (B) and *MtSVP2* (C) in leaves and dissected uppermost apical buds. Gene expression was determined using qRT-PCR and the data are shown as the mean ± SE of three biological replicates, which were normalized to *PDF2.* The data is presented relative to the highest value in both tissues for each gene. Samples were harvested at ZT2. The shaded area indicates the time point after prolonged cold exposure for 14 days.

A

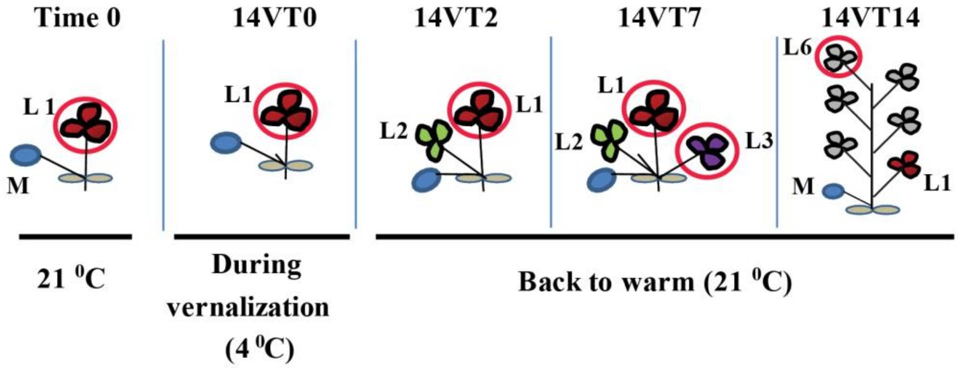
Time 0
14VT0
14VT2
14VT7
14VT14
L 1
M
L1
L2
L1
L1
L2
L3
L6
M
L1
21 ^{0}C
During vernalization (4 ^{0}C)
Back to warm (21 ^{0}C)

B

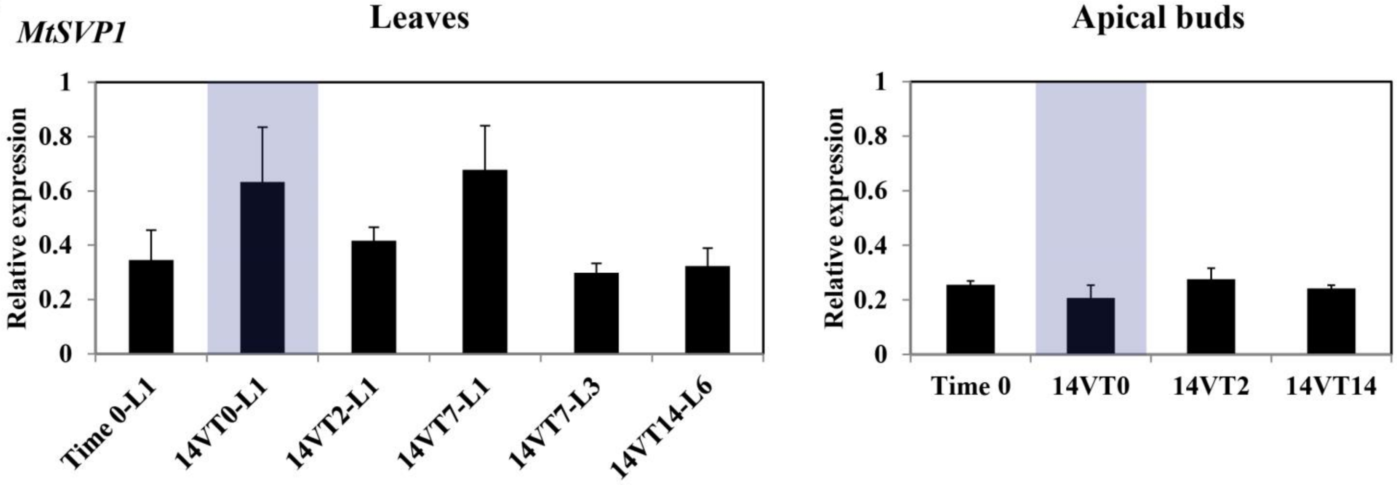
MtSVP1
Leaves
Apical buds
Relative expression
1
0.8
0.6
0.4
0.2
0
Time 0-L1
14VT0-L1
14VT2-L1
14VT7-L1
14VT7-L3
14VT14-L6
Time 0
14VT0
14VT2
14VT14

C

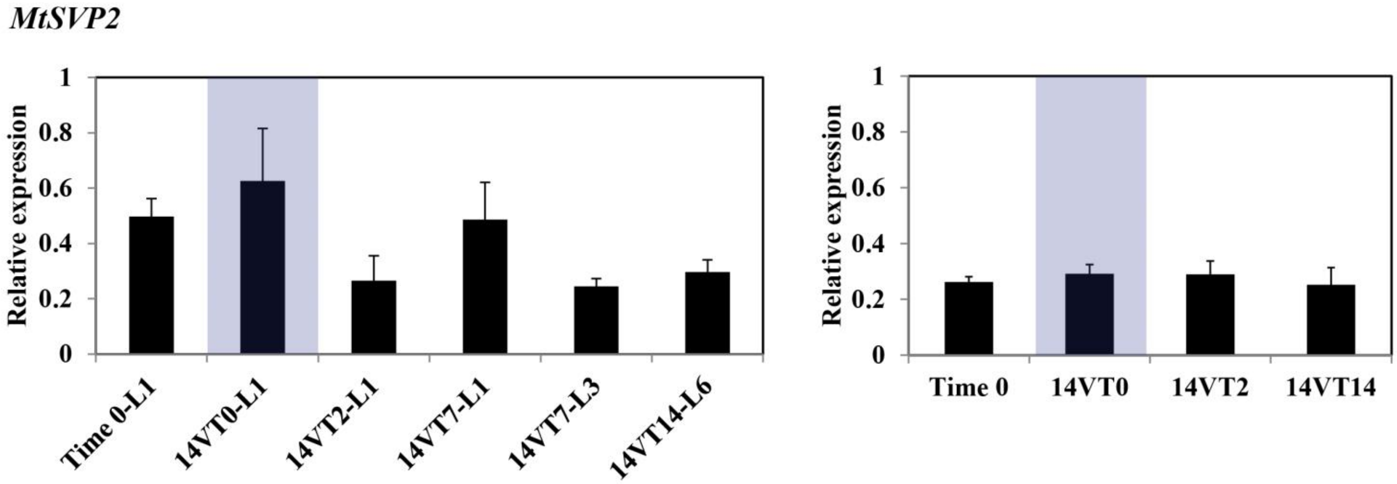
MtSVP2
Relative expression
1
0.8
0.6
0.4
0.2
0
Time 0-L1
14VT0-L1
14VT2-L1
14VT7-L1
14VT7-L3
14VT14-L6
Time 0
14VT0
14VT2
14VT14

Table S1. List of oligonucleotide primers (5' to 3') used in this study.

	Forward primer	Reverse primer	Use
MtSVP1	GGATATGGCTCGTGAGAAGATTCA	GGTTTAACCCTCGCACAAATCTAC	Cloning
MtSVP2	GGGATATGGCTCGTGAGAAGATTCA	TTCAGCCGGCATATGGTAGTCCCAAC	Cloning
MtSVP1	TGGAGGAGTTGAATCTGAGAACATGG	CAGCCAGCATATGGAAGCCCCAA	qRT-PCR
MtSVP2	GCGGGCATCATTAACGACAGAATGGTTG	GTCCCAACTTGAGCGATGTATCCGAACT	qRT-PCR
PDF2	GTGTTTTGCTTCCGCCGTT	CCAAATCTTGCTCCCTCATCTG	qRT-PCR
MtPIM	GCACAATGGGAGCACCCAAACCA	GCTTCCTCACGGTAATTGCCACCCA	qRT-PCR
TUBULIN	CTCCTAGCTTTGGTGACTTGAACC	TTTCCGGAGATCAGAGTTGAGTT	qRT-PCR
MtSVP2_NF11029	AGGTCACTTGAAATTGGGTTGGGCCGT	GTCGGTCGTTCTCTTCCATCAATTGCC	genotyping
MtSVP2_NF13617	GGTTGCGGGCATCATTAACGACA	TCAGCCGGCATATGGTAGTCTGC	genotyping
Tnt1-F	ACAGTGCTACCTCCTCTGGATG		genotyping

Table S2. Flowering time of T2 Arabidopsis transgenic plants from the overexpression of *MtSVP1* and *MtSVP2* in WT Col under LD conditions and classification of floral phenotypes. (A) The flowering time was measured as the total number of rosette and cauline leaves at flowering. The class of floral phenotype is based on the severity of floral defects as explained in (B). (B) The floral organ morphologies observed in transgenic plants transformed with *35S:MtSVP1* and *35S:MtSVP2* were classified into four categories: (1) same as WT; (2) weak = longer pedicels than WT, splayed arrangement of floral organs and lack of sepal abcission; (3) moderate = the weak phenotype including 5-7 anthers and 4-6 petals (†) and deformed siliques; and (4) extreme = up to three times increase on pedicel length compared with WT, more pronounced flower splaying, lack of sepal abscission, 6 sepals, up to 8 anthers and 9 petals (‡), sepaloid (green) petals, and enlarged and elongated carpels. [a]Numbers in this row represent increase in pedicel length relative to WT. A WT plant has 4 sepals, 4 petals, 6 stamens and 2 fused carpels.

A

	**Line**	**Flowering time (mean ± SE)**	**No. of transgenic T2 plants**	**Class of floral phenotype**
35S:MtSVP1 in Col	MJ244	15.1 ± 0.4	19	moderate
	MJ247	17.2 ± 0.6	18	moderate
	MJ257	18.7 ± 0.6	17	extreme
	MJ258	12.3 ± 0.3	18	same as wild type
WT Col		12.4 ± 0.4		
35S:MtSVP2 in Col	MJ291	14.5 ± 0.6	17	extreme
	MJ292	13.3 ± 0.4	15	moderate
	MJ293	14.7 ± 0.5	15	extreme
	MJ294	12.5 ± 0.4	15	same as wild type
	MJ295	12.1 ± 0.4	18	same as wild type
WT Col		10.6 ± 0.3		

B

	**WT**	**Weak**	**Moderate**	**Extreme**
Increased pedicel length[a]		2X	2X	2-3X
Flower splaying				
Lack of sepal abscission				
Change in anther and petal number			†	‡
Deformed siliques				
Sepaloid (green) petals				

Intensity of phenotype None Strong
